# The dopamine D_1_ receptor is expressed and induces CREB phosphorylation and MUC5AC expression in human airway epithelium

**DOI:** 10.1186/s12931-018-0757-4

**Published:** 2018-04-02

**Authors:** Nao Matsuyama, Sumire Shibata, Atsuko Matoba, Tada-aki Kudo, Jennifer Danielsson, Atsushi Kohjitani, Eiji Masaki, Charles W. Emala, Kentaro Mizuta

**Affiliations:** 1 Department of Dento-oral Anesthesiology, Tohoku University Graduate School of Dentistry, 4-1 Seiryo-machi, Aoba, Sendai, Miyagi 9808575 Japan; 2 Department of Oral Physiology, Tohoku University Graduate School of Dentistry, Sendai, Japan; 3 Department of Anesthesiology, College of Physicians and Surgeons of Columbia University, New York, NY USA; 4 Department of Dental Anesthesiology, Kagoshima University Graduate School of Medical and Dental Sciences, Kagoshima, Japan

**Keywords:** Dopamine D_1_ receptor, G_s_-coupled receptor, cAMP, CREB, MUC5AC

## Abstract

**Background:**

Dopamine receptors comprise two subgroups, G_s_ protein-coupled “D_1_-like” receptors (D_1_, D_5_) and G_i_-coupled “D_2_-like” receptors (D_2_, D_3_, D_4_). In airways, both dopamine D_1_ and D_2_ receptors are expressed on airway smooth muscle and regulate airway smooth muscle force. However, functional expression of the dopamine D_1_ receptor has never been identified on airway epithelium. Activation of G_s_-coupled receptors stimulate adenylyl cyclase leading to cyclic AMP (cAMP) production, which is known to induce mucus overproduction through the cAMP response element binding protein (CREB) in airway epithelial cells. We questioned whether the dopamine D_1_ receptor is expressed on airway epithelium, and whether it promotes CREB phosphorylation and MUC5AC expression.

**Methods:**

We evaluated the protein expression of the dopamine D_1_ receptor on native human airway epithelium and three sources of cultured human airway epithelial cells including primary cultured airway epithelial cells, the bronchial epithelial cell line (16HBE14o-), and the pulmonary mucoepidermoid carcinoma cell line (NCI-H292) using immunohistochemistry and immunoblotting. To characterize the stimulation of cAMP through the dopamine D_1_ receptor, 16HBE14o- cells and NCI-H292 cells were treated with dopamine or the dopamine D_1_ receptor agonists (SKF38393 or A68930) before cAMP measurements. The phosphorylation of CREB by A68930 in both 16HBE14o- and NCI-H292 cells was measured by immunoblot. The effect of dopamine or A68930 on the expression of *MUC5AC* mRNA and protein in NCI-H292 cells was evaluated by real-time PCR and immunofluorescence staining, respectively.

**Results:**

The dopamine D_1_ receptor protein was detected in native human airway epithelium and three sources of cultured human airway epithelial cells. Dopamine or the dopamine D_1_-like receptor agonists stimulated cAMP production in 16HBE14o- cells and NCI-H292 cells, which was reversed by the selective dopamine D_1_-like receptor antagonists (SCH23390 or SCH39166). A68930 significantly increased phosphorylation of CREB in both 16HBE14o- and NCI-H292 cells, which was attenuated by the inhibitors of PKA (H89) and MEK (U0126). Expression of *MUC5AC* mRNA and protein were also increased by either dopamine or A68930 in NCI-H292 cells.

**Conclusions:**

These results suggest that the activation of the dopamine D_1_ receptor on human airway epithelium could induce mucus overproduction, which could worsen airway obstructive symptoms.

## Background

Dopamine is a predominant catecholamine neurotransmitter in the mammalian central nervous system [1–4] but it also plays a role in modulating peripheral physiologic actions such as renal and cardiovascular functions through specific dopamine receptor subtypes expressed in peripheral organs and tissues [3, 5–8]. The dopamine receptors belong to the superfamily of G protein-coupled receptors (GPCR), and five different receptor subtypes (D_1_-D_5_) have been divided into two subgroups, the G_s_ protein-coupled “D_1_-like” receptors (D_1_, D_5_ subtypes) and the G_i_-coupled “D_2_-like” receptors (D_2_, D_3_, D_4_ subtypes) [3, 9]. Dopamine, by acting on the dopamine D_1_-like receptor, stimulates adenylyl cyclase activity to increase intracellular cyclic AMP (cAMP) levels [10], which stimulate cAMP-dependent protein kinase (PKA) [11]. PKA phosphorylates a range of target proteins including the cAMP response element binding protein (CREB) [12–14].

In airways, dopamine is localized in the lung [15], and acts as a neurotransmitter in addition to its role as a noradrenaline precursor [16]. Dopamine D_1_ and D_2_ receptors are expressed on lung alveolar type I cells, which line most of the alveolar surface, and contribute to lung fluid homeostasis [17]. In addition, either inhaled or intravenously administered dopamine has bronchodilatory effects in human healthy and asthmatic subjects [18]. We have previously shown that dopamine D_1_ and D_2_ receptors are expressed on airway smooth muscle itself, and that the dopamine D_1_ receptor modulates airway smooth muscle tone through adenylyl cyclase/cAMP production [19, 20], which would favor airway relaxation in asthmatics. Although, the dopamine D_2_ receptor was not detected on airway epithelial tissue [19], the functional expression of the dopamine D_1_-like receptor on airway epithelium remains poorly characterized.

In respiratory diseases including asthma, COPD, and cystic fibrosis, mucus hypersecretion is a recognized component of the pathophysiology. Airway epithelium is the predominant source of mucus, which contributes to airway narrowing and obstruction. MUC5AC, which is induced by phosphorylation of CREB [21, 22], is predominantly expressed in respiratory epithelium and constitutes 95–98% of the mucin secreted in the human airway [23]. Interestingly, the dopamine D_1_-like receptor agonist SKF83959 significantly exacerbated bronchial mucus production in ovalbumin-sensitized mice [24], which would in theory, therapeutically contrast with its direct relaxation of airway smooth muscle [20]. Similar contrasting findings have been reported with G_s_-coupled β_2_-aderenoceptor agonists, which are widely used as bronchodilators, but have been reported to increase mucin production via activation of β_2_-aderenoceptors on airway epithelial cells [25]. These findings led us to hypothesize that functional dopamine D_1_-like receptors are expressed on airway epithelium and promote mucus production through cellular cAMP’s activation of the PKA-CREB-MUC5AC axis.

In the present study, protein expression of the dopamine D_1_-like receptor was examined in native human airway epithelial tissue and cultured human airway epithelial cells. In addition, effects of the dopamine D_1_ receptor on cAMP production, CREB phosphorylation, and MUC5AC expression were assessed to confirm their physiological role in airway epithelium.

## Methods

### Materials

Protease inhibitor cocktail III was purchased from EMD Millipore (Billerica, MA). Antibiotic-antimycotic mix, DMEM/F-12 medium, fetal bovine serum (FBS), and RPMI-1640 medium were purchased from Thermo Fisher Scientific (Waltham, MA). A68930 and SCH39166 were purchased from Tocris Bioscience (Bristol, UK). All other chemicals were obtained from Sigma-Aldrich (St. Louis, MO) unless otherwise stated.

### Cell culture

Primary cultured normal human bronchial epithelial cells (CC-2541; Lonza, Walkersville, MD) were grown in Clonetics™ BEGM BulletKit (CC-3170, Lonza) supplemented with the following growth supplements: bovine pituitary extract, hydrocortisone, human epidermal growth factor, epinephrine, transferrin, insulin, retinoic acid, triiodothyronine, and gentamicin/amphotericin-B at the concentrations recommended by the manufacturer. 16HBE14o- cells, a human bronchial epithelial cell line which was kindly gifted from Dr. Tilla S. Worgall (Columbia University, New York NY), were grown in minimal essential medium supplemented with 10% FBS and 200 μg/ml geneticin (G418). NCI-H292 cells (CRL-1848; American Type Culture Collection, Manassas, VA), a human pulmonary muco-epidermoid carcinoma cell line, were cultured in RPMI-1640 medium containing 5% FBS. Primary cultured human airway smooth muscle cells (HASM; cc-2576, Lonza) were grown in DMEM/F12 culture medium, supplemented with 10% FBS and an antibiotic-antimycotic mix (100 units/ml penicillin G sodium, 100 μg/ml streptomycin sulfate, 0.25 μg/ml amphotericin B). All the cells were incubated at 37°C in humidified 95% air/5% CO_2_.

### Preparation of human trachea

Studies were approved by Columbia University’s Institutional Review Board (IRB) and deemed not human subjects research under 45 CFR 46. Human trachea was obtained from discarded regions of healthy donor lungs harvested for lung transplantation at Columbia University. Human tissue was transported to the laboratory in cold (4 °C) M199 cell culture media. The exterior of human trachea was carefully dissected free of adherent connective tissue under a microscope. The tissue sample was used for immunohistochemistry and immunoblot.

### Immunohistochemistry

Human tracheal rings were fixed with 4% paraformaldehyde/1% glutaraldehyde in 0.1 M phosphate buffer for 4 h at 4°C and were then dehydrated through a graded ethanol series. The tracheal rings were embedded in paraffin and cut into 10-μm-thick sections. The tracheal ring sections were deparaffinized in xylene and rehydrated in descending grades of alcohol. Heat-mediated antigen retrieval was performed in Tris-EDTA buffer (10 mM Tris-base and 1 mM EDTA, pH 9.0) for 2 min using a pressure cooker. Endogenous peroxidase activity was blocked with 3% H_2_O_2_ for 15 min. Sections were blocked with 10% normal goat serum in phosphate-buffered saline with 0.1% Triton X-100 (PBST) for 30 min, then avidin-biotin blocking was performed as previously described [26]. The slides were kept in humidified chamber with primary antibody against the dopamine D_1_ receptor protein (rabbit monoclonal 1:2000; 2192–1, Epitomics, Burlingame, CA) or dopamine D_5_ receptor protein (rabbit polyclonal 1:2000; sc-25,650, Santa Cruz Biotechnology, Santa Cruz, CA) in 2% normal goat serum in PBST for overnight at 4 °C. The immunoreactivity of these antibodies directed against the dopamine D_1_ or D_5_ proteins were previously confirmed in control tissues during immunohistochemistry [20]. A parallel tracheal ring section was incubated with an isotype-specific rabbit IgG antibody (Thermo Fisher Scientific) as a negative control. The slides were then washed three times with PBST and primary antibodies were detected using biotinylated anti-rabbit antibodies (Vector Laboratories, Burlingame, CA) at a concentration of 1:200. After incubation with ABC-HRP complex (Vector Laboratories) for 30 min, the antigen-antibody complex was visualized with the peroxidase substrate kit (DAB) (SK-4100, Vector Laboratories). The sections were counterstained with hematoxylin (Vector Laboratories), dried, dehydrated in ascending grades of alcohol, and cover slipped using Poly-mount mounting medium (Polysciences, Warrington, PA).

### Immunoblot analysis

Freshly dissected native human airway epithelium was homogenized (Tekmar Ultra Turrax T25 high-speed homogenizer set at top speed for 30 s) in cold (4°C) buffer (50 mM Tris, 10 mM HEPES, pH 7.4, 1 mM EDTA with a 1:200 dilution of protease inhibitor cocktail III). The homogenate was filtered through 125-μm Nitex mesh and centrifuged twice at 500 *g* for 15 min. The supernatant was transferred into new tubes and centrifuged at 50,000 *g* for 30 min at 4°C. The final membrane pellet was resuspended in the same buffer for protein concentration determinations and stored at − 80°C.

For analysis of dopamine D_1_ receptor expression, confluent cultures of either primary cultured normal human bronchial epithelial cells, 16HBE14o- cells, NCI-H292 cells, or primary cultured human airway smooth muscle cells were rinsed with ice-cold phosphate-buffered saline (PBS), and mechanically scraped from the surface of the T75 culture flask in the presence of protease inhibitor cocktail III. Cells were pelleted (500 *g*, 10 min, 4 °C) and lysed in ice-cold lysis buffer [20 mM Tris-HCl, pH 7.5, 150 mM NaCl, 1 mM Na_2_EDTA, 1 mM EGTA, 1% Nonidet P-40, 1% sodium deoxycholate, 2.5 mM sodium pyrophosphate, 1 mM β-glycerophosphate, 1 mM Na_3_VO_4_, 1 μg/ml leupeptin, 1 mM phenylmethanesulfonyl fluoride, 1:200 dilution of protease inhibitor cocktail III]. Lysed cells were centrifuged (15,000 *g*, 15 min, 4 °C) and an aliquot of the supernatant was subjected to protein analysis and storing at − 80 °C. For analysis of CREB phosphorylation, 16HBE14o- cells or NCI-H292 cells were serum-starved for 24 h, and then treated with dopamine D_1_ receptor agonist A68930 (1 μM) for indicated times (5–60 min). In separate experiments, NCI-H292 cells were initially pretreated with 10 μM H89 (PKA inhibitor; 30 min) or 5 μM U0126 (MEK inhibitor; 120 min) before treatment of the cells with A68930 (1 μM; 20 min). After treatment, the cells were washed twice with ice-cold PBS, and lysed in ice-cold RIPA buffer (Cell signaling Technology (CST), Danvers, MA) supplemented with 1 mM phenylmethanesulfonyl fluoride and a 1:200 dilution of protease inhibitor cocktail III. Each lysed cell sample was harvested and centrifuged at 15000 *g* for 15 min at 4 °C, and an aliquot of the supernatant was subjected to protein analysis. The protein concentration of each sample was determined using Pierce BCA reagents (Thermo Fisher Scientific), using BSA as a control, and samples were stored at − 80 °C. Each sample was solubilized by heating at 95 °C for 10 min in sample buffer (final concentrations: 50 mM Tris HCl pH 6.8, 2.5% SDS, 6% glycerol, 2.5% 2-mercaptoethanol, and bromophenol blue) before use. Lysates were electrophoresed (10% Mini-Protean TGX™ precast gel; Bio-Rad, Hercules, CA) and transferred to PVDF membranes using a Trans-Blot Turbo™ transfer system (Bio-Rad). The PVDF membrane was blocked for 1 h at room temperature with 5% membrane blocking agent (RPN418; GE Healthcare, Waukesha, WI) in Tris-buffered saline with 0.1% Tween 20 (TBST). Membranes were then probed with antibodies directed against the dopamine D_1_ receptor protein (rabbit monoclonal 1:1000; 2192–1, Epitomics) or the dopamine D_5_ receptor protein (rabbit polyclonal 1:500; sc-25,650, Santa Cruz Biotechnology) overnight at 4 °C. For the CREB phosphorylation study, the membranes were probed with antibodies directed against the anti-phospho CREB (rabbit monoclonal 1:1000; CST #9198), or anti-CREB (rabbit monoclonal 1:1000; CST #9197) overnight at 4 °C. After washing three times with TBST, membranes were incubated for 1 h at room temperature with HRP-labeled secondary anti-rabbit antibodies (1:5000; GE Healthcare, NA934V). The signals from the immunoreactive bands were detected by ECL Prime (GE Healthcare) and the signal was captured using a chemiluminescent image analyzer (LAS 4000 Mini; GE Healthcare). The same PVDF membranes were stripped and reprobed with the antibody against the GAPDH protein (rabbit monoclonal 1:2000, CST #5174) to demonstrate the variation in protein loading on the gels. For analysis of CREB phosphorylation, the band intensities were measured using Image J software (NIH) and were expressed as a ratio of the phosphorylated/total CREB protein.

### cAMP assays

Cyclic AMP (cAMP) production in 16HBE14o- and NCI-H292 cell lines was measured using a HitHunter™ cAMP Assay for Small Molecules kit (DiscoverX, Fremont, CA) according to the manufacturer’s instructions. Briefly, the cells grown in white-walled 96-well plates and were washed twice with warm PBS (37 °C). The cells were incubated with dopamine (1 μM), or the dopamine D_1_-like receptor agonists (A68930 or SKF38393) (1 μM) for 20 min at 37 °C. The concentration and duration of the dopamine D_1_-like receptor agonists used in this study was determined based on previous studies [20, 27]. In separate experiments, the cells were pretreated with the dopamine D_1_-like receptor antagonists [SCH23390 (1 μM) or SCH39166 (1 μM)] or vehicle (PBS) for 30 min followed by incubation with A68930 (1 μM) for 20 min at 37 °C. Then the cAMP antibody reagent followed by the cAMP working solution (mixture of enzyme donor/lysis buffer/Emerald II/Galacton) was added to each well and incubated for 60 min at room temperature. Cells were further incubated with the enzyme acceptor reagent for 3 h at room temperature, and luminescence signals were detected using a multimode microplate reader (Appliskan, Thermo Fisher Scientific). The data from the triplicate wells were averaged for each sample.

### Preparation of cigarette smoke extract

Cigarette smoke extract (CSE) was prepared by a modification of a previously published method [28]. Smoke of four commercial filtered cigarettes (Marbolo Red, Philip Morris, Richmond, VA; 1.0 mg nicotine; 12 mg Tar) was bubbled through 50 mL of PBS using a vacuum pump. This 100% CSE solution was adjusted to pH 7.4 and filtered through a 0.2 μm-pore filter to remove bacteria and large particles.

### Cell viability assay

NCI-H292 cell viability after 48 h treatment with CSE (10% or 20%) was measured using an MTT assay kit (Cayman Chemical, Ann Arbor, MI) according to the manufacturer’s instructions. Briefly, NCI-H292 cells were seeded in a 96-well plate (5000 cells/well) in RPMI-1640 medium containing 0.5% FBS for 24 h. Then, the cells were incubated with or without CSE (10% or 20%) for 48 h in a final volume of 100 μl/well. Ten microliters of MTT reagent were added to each well and the cells were re-incubated for 4 h at 37 °C in a CO_2_ incubator. The medium was aspirated, and 100 μl of the crystal dissolving solution were added to each well. The absorbance of each sample was measured at 570 nm using a multimode microplate reader (Appliskan; Thermo Fisher Scientific). The results were expressed as a percentage of surviving cells over control (no treatment) cells.

### Quantitative RT-PCR

Total RNA was extracted from cultured NCI-H292 cells after 48 h treatment with dopamine (1 μM), A68930 (1 μM), isoproterenol (1 μM), or CSE (10%) using the RNeasy Mini Kit (QIAGEN, Valencia, CA). Total RNA was transcribed into cDNA using the ReverTra Ace qPCR RT Kit (Toyobo, Osaka, Japan) in accordance with the manufacturer’s instructions. Quantitative real-time PCR on the CFX96 Real-Time PCR Detection System (Bio-Rad) was performed using Thunderbird SYBR qPCR kit (Toyobo) according to manufacturer’s instruction. Primer sequences for *MUC5AC* and *GAPDH* were shown in Table 1. The specificity of amplification was confirmed by melting curve analysis. The Ct value determined by the CFX manager Software (Bio-Rad) for all samples was normalized to the housekeeping gene *GAPDH*, and the relative fold induction against untreated controls was computed by the comparative Ct (ΔΔCt) method.

**Table 1:** Primer sequences

Target	Sequence of Primer	Amplicon size (bp)
Human MUC5AC	FP: 5′- GGA GGA AGC TGG CCC TGC TCT GG-3’	116
	RP: 5′- AGA GAG GGC AGG GTG GTG CTT GT-3’	
Human GAPDH	FP: 5′- CCA GGG CTG CTT TTA ACT CTG GTA AAG TGG ATA-3’	173
	RP: 5′- CAT CGC CCC ACT TGA TTT TGG AGG GA −3’	

*FP* forward primer, *RP* reverse primer

### Immunofluorescence staining

Immunofluorescence staining of MUC5AC protein in NCI-H292 cells was carried out according to the previously described method [29] with some modifications. Briefly, NCI-H292 cells were seeded on an 8-chamber microscope slide and serum-starved for 24 h. After starvation, cells were exposed to A68930 (1 μM), dopamine (1 μM), or CSE (10%) for 48 h. Cells were fixed with 4% paraformaldehyde for 15 min at room temperature and washed 3 times with PBS. After permeabilization (0.2% Triton X-100 in PBS for 5 min) and blocking (1% bovine serum albumin in 0.1% Triton X-100 in PBS for 15 min), cells were incubated with Alexa Fluor 488-conjugated MUC5AC antibody (sc-21,701 AF488, Santa Cruz Biotechnology) overnight at 4C. After cells were washed twice with PBS, the slide was coverslipped with ProLong gold antifade-reagent with DAPI (Thermo Fisher Scientific), and visualized with an inverted fluorescent microscope (DMI-4000; Leica Microsystems, Wetzlar, Germany). Digitized images were captured with MetaMorph software (Molecular Devices, Sunnyvale, CA). When capturing the images, we kept constant the duration of image capture (300 ms), the image intensity gain, the image enhancement, and the image black level among the samples.

### Statistical analysis

The data were analyzed with two-tailed paired Student’s t-test when comparing means between two groups or repeated measures of ANOVA followed by Bonferroni post test when comparing multiple groups using GraphPad Prism 6 for Mac OS X software (GraphPad Software, La Jolla, CA). Data are presented as mean ± SEM; *P* < 0.05 was considered significant.

## Results

### Immunohistochemical detection of dopamine D_1_-like receptor expression in human tracheal epithelium

**Fig. 1:**
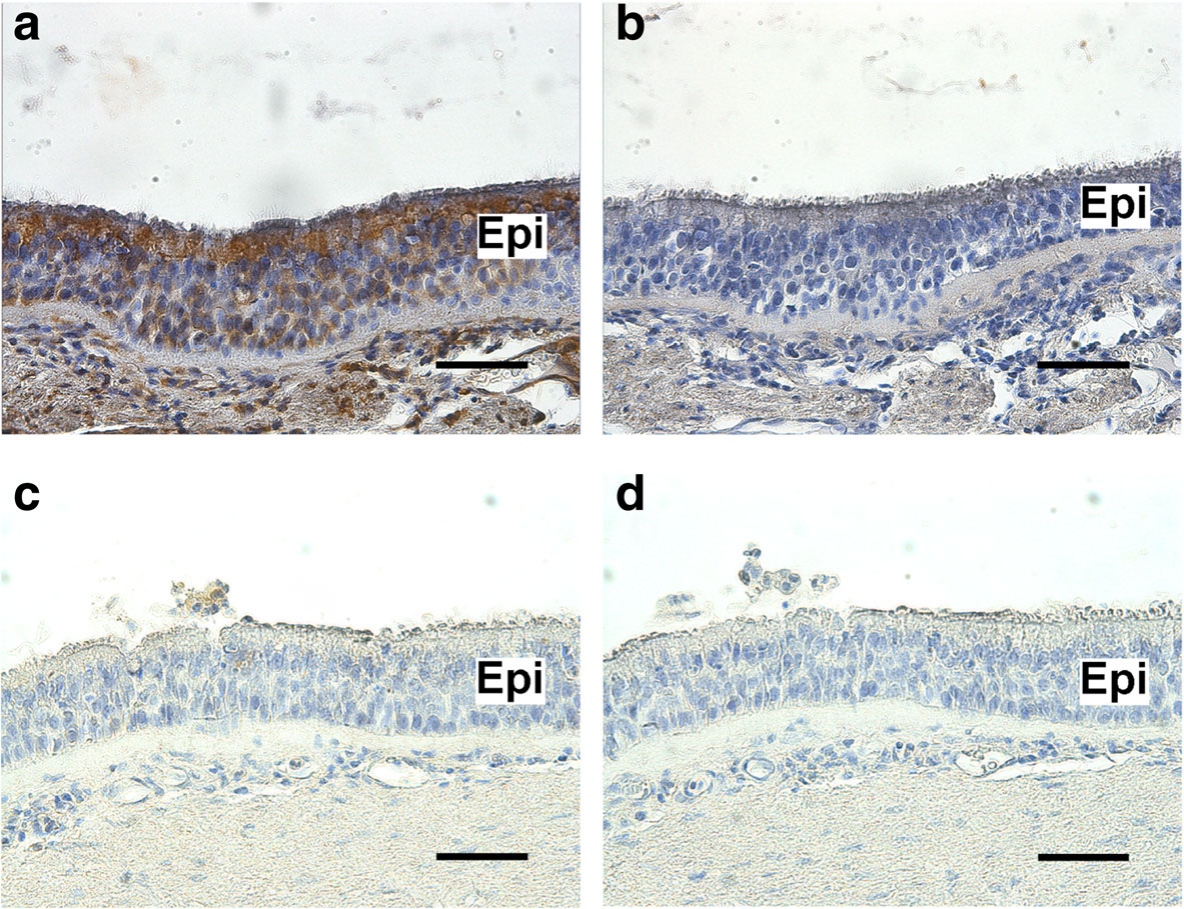
Immunohistochemical detection of dopamine D_1_-like receptor expression in human tracheal epithelium. **a** and **c** Representative immunohistochemical staining of dopamine D_1_ receptor (**a**) and dopamine D_5_ receptor (**c**) in paraformaldehyde/glutaraldehyde-fixed human tracheal epithelium. **b** and **d** anti-rabbit IgG isotype negative control in serial section of human tracheal epithelium. All sections were counterstained with hematoxylin. Calibration bars: 50 μm. Epi, airway epithelium. Images are representative of at least 3 independent immunohistochemical analyses from human trachea

Initially, we examined the protein expression of the dopamine D_1_ and D_5_ receptor in human tracheal epithelium by immunohistochemistry. Light microscopic immunohistochemical staining for the dopamine D_1_ receptor was observed throughout the epithelial layer of human trachea (indicated by brown color) (Fig. 1a). In contrast, no staining of the dopamine D_5_ receptor was detected in the airway epithelial layer (Fig. 1c). Consecutive sections exposed to rabbit IgG isotype-specific control antibodies yielded no staining (Fig. 1b and d).

### Immunoblot analysis of the dopamine D_1_ receptor in human airway epithelium

**Fig. 2:**
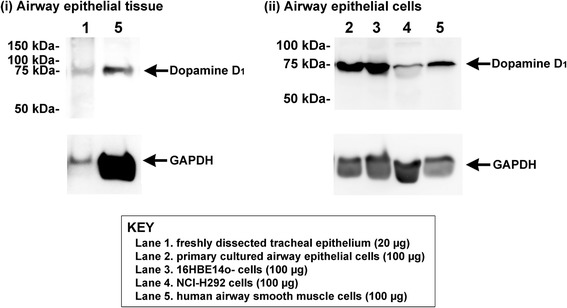
Representative immunoblot analyses using antibodies against the dopamine D_1_ receptor using total protein prepared from freshly dissected native human tracheal epithelium (20 μg), primary cultured human airway epithelial cells (100 μg), the human bronchial epithelial cell line (16HBE14o-) (100 μg), the human pulmonary mucoepidermoid carcinoma cell line (NCI-H292) (100 μg), and human airway smooth muscle cells (positive control) (100 μg). Reprobing of blots for GAPDH was performed to demonstrate relative lane loading. Each image is representative of at least 3 independent immunoblots

We further examined whether the dopamine D_1_ receptor protein was expressed in human airway epithelial tissue and cells by immunoblot. A single immunoreactive band of the appropriate molecular mass for the dopamine D_1_ receptor (75 kDa) was identified in freshly dissected human tracheal epithelium, human pulmonary muco-epidermoid carcinoma cells (NCI-H292 cells), human bronchial epithelial cells (16HBE14o- cells), primary cultured human bronchial epithelial cells, and primary cultured human airway smooth muscle cells (positive control) (Fig. 2). These results suggest that the dopamine D_1_ receptor is expressed on human airway epithelium of both trachea and bronchi as well as human airway smooth muscle.

### Dopamine D_1_ receptor agonist-induced cAMP activity in 16HBE14o- cells and NCI-H292 cells

**Fig. 3:**
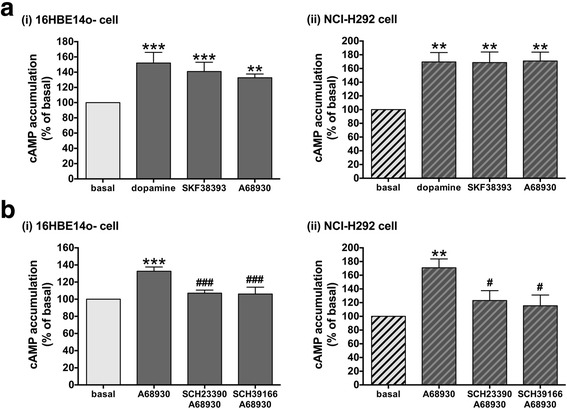
**a** The effects of dopamine (1 μM) or the dopamine D_1_ receptor agonists (A68930 or SKF38393; 1 μM respectively) on cAMP production in cultured (i) 16HBE14o- cells and (ii) NCI-H292 cells. *n* = 6. **b** The effect of dopamine D_1_-like receptor selective antagonists SCH23390 or SCH39166 on A68930-stimulated cAMP production in cultured (i) 16HBE14o- cells and (ii) NCI-H292 cells. Cells were pretreated with SCH23390 (1 μM) or SCH39166 (1 μM) for 30 min prior to A68930 (1 μM) treatment for 20 min. *n* = 6. Data represent means ± SEM. **P < 0.01 and ***P < 0.001 compared with basal. ^#^*P <* 0.05 and ^###^*P <* 0.001 compared with A68930 alone

The dopamine D_1_ receptor induces production of cAMP through the stimulation of adenylyl cyclase, which is activated by the G_s_ protein [30]. Therefore, we examined whether activation of the dopamine D_1_ receptor increases intracellular cAMP levels in human airway epithelial cells (16HBE14o- cells and NCI-H292 cells). Dopamine (1 μM) as well as dopamine D_1_-like receptor agonists (SKF38393 or A68930; 1 μM) significantly increased intracellular cAMP levels in 16HBE14o- cells (dopamine; *P* < 0.001, SKF38393; *P* < 0.001, A68930; *P* < 0.01, *n* = 6) and NCI-H292 cells (dopamine; *P* < 0.01, SKF38393; *P* < 0.01, A68930; *P* < 0.01, *n* = 6) (Fig. 3a). Cyclic AMP production induced by A68930 (1 μM) was significantly reversed by pretreatment with the dopamine D_1_ receptor antagonists SCH23390 (1 μM) (16HBE14o- cells: *P <* 0.001, *n* = 6; NCI-H292 cells: *P <* 0.05, *n* = 6) or SCH39166 (1 μM) (16HBE14o- cells: *P <* 0.001, n = 6; NCI-H292 cells: *P <* 0.05, n = 6) (Fig. 3b).

### Dopamine D_1_ receptor agonist-induced CREB phosphorylation in 16HBE14o- cells and NCI-H292 cells

**Fig. 4:**
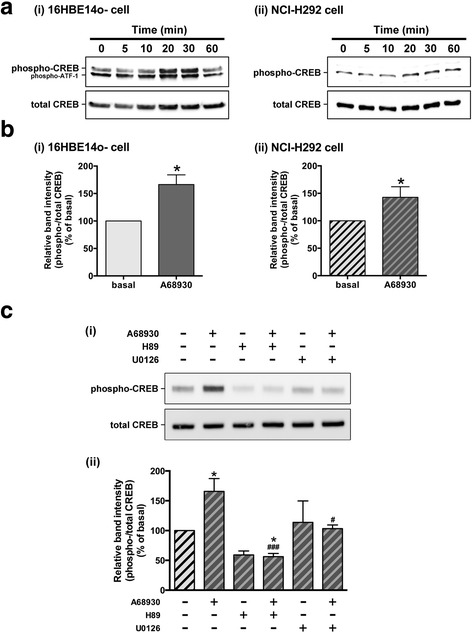
Effects of the dopamine D_1_ receptor agonist A68930 on the phosphorylation of CREB in cultured airway epithelial cells. Cells were stimulated with A68930 (1 μM), and subsequently cell lysates were processed to detect phosphorylated (upper panels) and total (lower panels) levels of CREB by immunoblot. **a** Representative immunoblot analyses of the time-course effect of the dopamine D_1_ receptor agonist A68930 (1 μM) on CREB phosphorylation in cultured (i) 16HBE14o- cells and (ii) NCI-H292 cells. Phosphorylated activating transcription factor 1 (ATF-1) is a CREB family member that is also recognized by the phospho-CREB antibody and closely correlates with CREB [13]. **b** Effect of A68930 (1 μM, 20 min) on CREB phosphorylation in (i) 16HBE14o- cells (*n* = 3) and (ii) NCI-H292 cells (*n* = 7). **c** Effects of inhibitors of PKA or MEK on A68930-stimulated CREB phosphorylation in NCI-H292 cells (*n* = 4). Cells were pretreated with the PKA inhibitor H89 (10 μM, 30 min), or the MEK inhibitor U0126 (5 μM, 120 min) before treatment with A68930 (1 μM, 20 min). Data represent means ± SEM. *P < 0.05 compared with basal. ^#^*P <* 0.05 and ^###^*P <* 0.001 compared with A68930 alone

The increase of cAMP after dopamine D_1_ receptor activation induces the activation of protein kinase A (PKA), which induces phosphorylation of CREB [14]. MEK-ERK signaling also contributes to CREB phosphorylation [13]. In addition, the dopamine D_1_ receptor activates MEK-ERK signaling through β-arrestin [31]. Therefore, we examined whether the dopamine D_1_ receptor agonist A68930 phosphorylates CREB through PKA and/or MEK in 16HBE14o- cells and NCI-H292 cells. A68930 (1 μM, 20 min) significantly increased phosphorylation of CREB in 16HBE14o- cells (*P* < 0.05, *n* = 3) and NCI-H292 cells (*P* < 0.05, *n* = 7) (Fig. 4a and b). The phosphorylation reached maximal levels at 20–30 min and then slowly declined to basal levels within 60 min in 16HBE14o- cells, while the increased phosphorylation was maintained at 60 min in NCI-H292 cells. To confirm that A68930 phosphorylates CREB through PKA or MEK, NCI-H292 cells were pretreated with the PKA inhibitor H89 (10 μM; 30 min) or the MEK inhibitor U0126 (5 μM; 120 min). A68930 (1 μM; 20 min)-stimulated CREB phosphorylation in NCI-H292 cells was significantly inhibited by H89 (*P* < 0.001, *n* = 4) or U0126 (*P* < 0.05, *n* = 4) (Fig. 4c). These results confirm that the dopamine D_1_ receptor agonist-induced phosphorylation of CREB proceeds through both PKA and MEK/ERK signaling.

### Dopamine D_1_ receptor agonist-induced *MUC5AC* mRNA expression in NCI-H292 cells

**Fig. 5:**
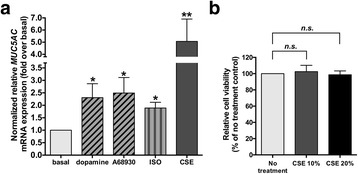
**a** Effect of dopamine D_1_ receptor agonists on *MUC5AC* mRNA expression in NCI-H292 cells. The cells were treated for 48 h with dopamine (1 μM), the dopamine D_1_ receptor agonist A68930 (1 μM), the G_s_ protein-coupled β_2_ adrenoceptor agonist isoproterenol (ISO; 1 μM), or cigarette smoke extract (CSE; 10%: positive control). Data represent means ± SEM. *P < 0.05, **P < 0.01 compared with basal. *n* = 14. **b** NCI-H292 cell viability analysis with MTT assay after 48 h treatment with CSE (10 or 20%); *n* = 13. Data are shown as percentages of absorbance at 570 nm compared with no treatment control and represent means ± SEM. *n.s.* compared with no treatment control

CREB was previously shown to mediate the transcriptional regulation of MUC5AC in airway epithelial cells including NCI-H292 cells [21, 22, 32]. We examined whether dopamine or the dopamine D_1_ receptor agonist A68930 induces *MUC5AC* mRNA expression in NCI-H292 cells. Dopamine (1 μM), A68930 (1 μM), and cigarette smoke extract (CSE) (10%) significantly increased *MUC5AC* mRNA expression in NCI-H292 cells. The G_s_ protein-coupled β_2_ aderenoceptor agonist isoproterenol (1 μM) also significantly induced *MUC5AC* mRNA expression (Fig. 5a). Previous studies in airway epithelial cells have employed final CSE concentrations ranging from 1 to 30% [33, 34]. MTT cell viability analyses confirmed that 48 h treatment of NCI-H292 cells with 10% CSE or even higher concentrations (20%) of CSE did not reduce NCI-H292 cell viability, suggesting that treatment with CSE at 10% or 20% has no cytotoxicity (Fig. 5b).

### Dopamine D_1_ receptor agonist-induced MUC5AC protein expression in NCI-H292 cells

**Fig. 6:**
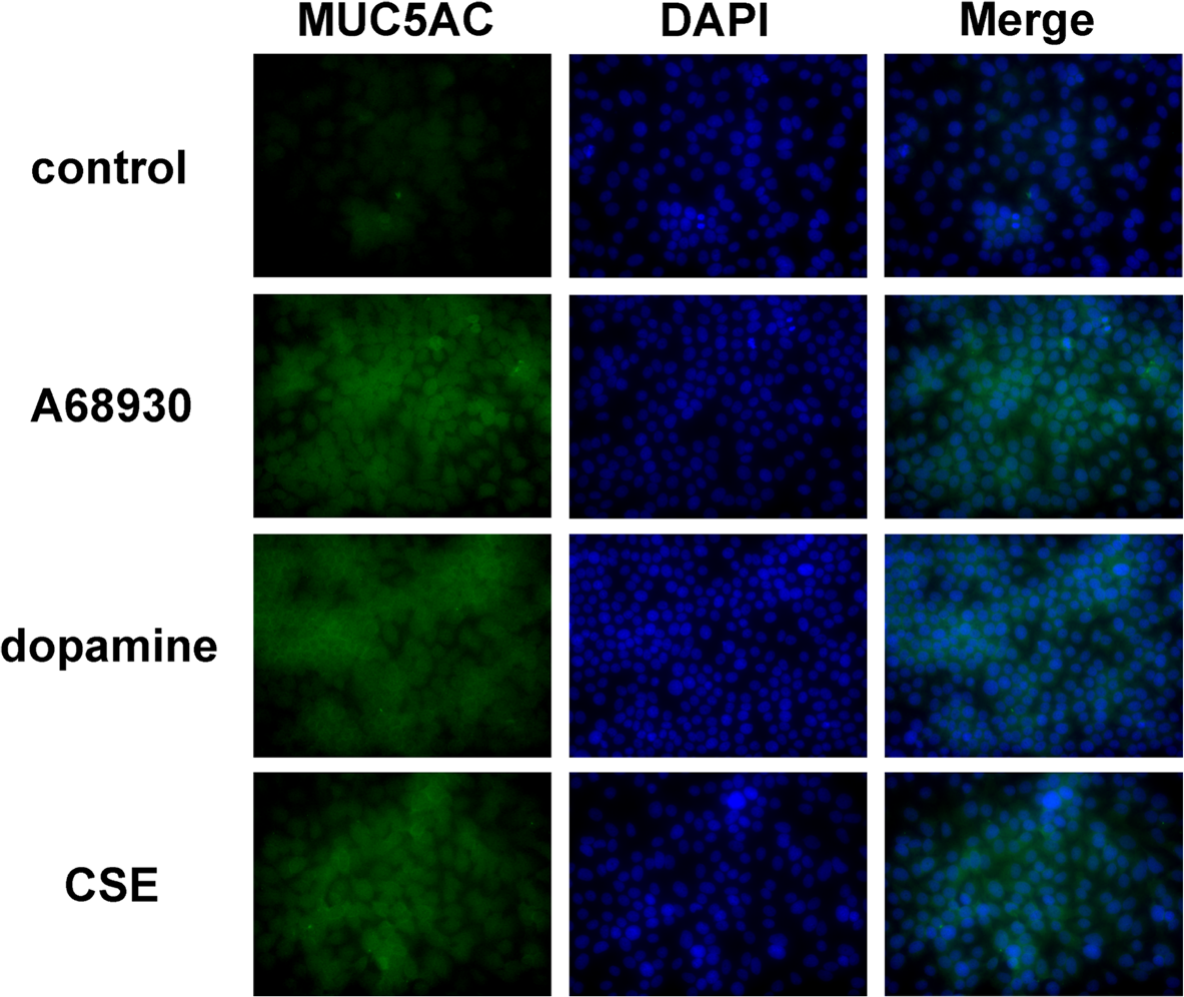
Representative images of immunofluorescent staining of NCI-H292 cells with an Alexa Fluor 488-conjugated mouse monoclonal antibody against human MUC5AC (green). Cells were incubated in the presence or absence of the dopamine D_1_ receptor agonist A68930 (1 μM), dopamine (1 μM), or CSE (10%: positive control) for 48 h before staining. Nuclei were stained with DAPI (blue). Images are representative of 3 independent immunofluorescence stainings

We further investigated the effect of the dopamine D_1_ receptor agonist A68930 or dopamine on MUC5AC protein expression using an immunofluorescent assay. Consistent with the mRNA data of *MUC5AC*, MUC5AC protein expression in NCI-H292 cells were increased by dopamine (1 μM), A68930 (1 μM), and CSE (10%) (Fig. 6). These results suggest that activation of dopamine D_1_ receptor stimulates MUC5AC expression.

## Discussion

The primary findings of the present study are that functional dopamine D_1_ receptors are expressed in human airway epithelium. Activation of the dopamine D_1_ receptor stimulated cAMP production, CREB phosphorylation, and mRNA and protein expression of MUC5AC in human airway epithelial cells.

In airways, we have previously reported that both dopamine D_1_ and D_2_ receptors are expressed on airway smooth muscle itself, and regulate airway smooth muscle force [19, 20]. In the present study, protein expression of the dopamine D_1_ receptor on human airway epithelium was identified by immunohistochemistry, while the dopamine D_5_ receptor protein was not detected. Immunoblot analyses further confirmed that the dopamine D_1_ receptor is expressed in freshly dissected native human tracheal epithelium, primary cultured human bronchial epithelial cells and cell lines derived from airway epithelium (16HBE14o- cells and NCI-H292 cells). Since the dopamine D_2_ receptor was not expressed on airway epithelium [19], the dopamine D_1_ receptor would be the predominant dopamine receptor subtype expressed on human airway epithelium.

Thereafter, we further investigated whether functional dopamine D_1_ receptors could modulate airway epithelial function. The activation of G_s_-coupled receptors including the dopamine D_1_ receptor and β_2_-adrenoceptor stimulates cAMP accumulation via activation of adenylyl cyclase [3, 35, 36]. In accordance with these previous findings, we demonstrated that dopamine or the dopamine D_1_ receptor agonists (SKF38393 or A68930) stimulated cAMP production in 2 different cell lines of human airway epithelial cells (16HBE14o- cells and NCI-H292 cells), which was significantly reversed by pretreatment the cells with the dopamine D_1_-like receptor antagonists (SCH23390 or SCH39166). These findings suggest that stimulation of the dopamine D_1_ receptor on human airway epithelial cells induces intracellular cAMP accumulation.

A major signaling target of cAMP is PKA, which can translocate to the nucleus and subsequently phosphorylate CREB. The present study demonstrated that the dopamine D_1_ receptor agonist A68930 induced phosphorylation of CREB in 16HBE14o- cells and NCI-H292 cells. This phosphorylation reached maximum levels at 20–30 min which is consistent with our findings that A68930 significantly induced cAMP accumulation at 20 min in 16HBE14o- cells and NCI-H292 cells. Furthermore, A68930-induced CREB phosphorylation was blocked by the PKA inhibitor H89, suggesting that activation of the dopamine D_1_ receptor induces CREB phosphorylation through classical cAMP-PKA signaling. Other principal signaling cascades responsible for CREB phosphorylation include the MEK-ERK pathway [13]. Previous studies suggested that the dopamine D_1_ receptor activates MEK-ERK signaling through β-arrestin [31]. In the present study, the MEK inhibitor U0126 significantly inhibited dopamine D_1_ receptor-mediated CREB phosphorylation. Collectively, activation of dopamine D_1_ receptor induces CREB phosphorylation through both cAMP/PKA and MEK/ERK signaling.

CREB increases the transcription of MUC5AC in NCI-H292 cells [21]. Our findings demonstrated that dopamine or the dopamine D_1_ receptor agonist A68930 induced expression of *MUC5AC* mRNA and MUC5AC protein, the most prominent mucin in airways. These results were consistent with the previous findings from Gong et al. [24] that the dopamine D_1_-like receptor agonist SKF83959 significantly exacerbated bronchial mucus production. They also showed that the dopamine D_1_-like receptor antagonist SCH23390 attenuated mucus production in the ovalbumin-sensitized mice, although they speculated that attenuated mucus production after blockade of dopamine D_1_-like receptor signaling by SCH23390 was predominantly mediated by decreased IL-17 secretion. In contrast, our results suggest that Gs-coupled dopamine D_1_ receptor signaling could contribute to MUC5AC expression through cAMP/CREB pathways without involving inflammatory mediators.

Although activation of the dopamine D_1_ receptor expressed on airway smooth muscle induced airway relaxation [20], the present study suggests that activation of the dopamine D_1_ receptor on airway epithelium could worsen asthma symptoms by mucus overproduction. These paradoxical effects of dopamine D_1_ receptor activation on airways would hamper the clinical use of a dopamine D_1_ receptor agonist as a novel treatment option of asthma and COPD. Similar paradoxical findings have been reported in other G_s_-coupled receptor (e.g. β_2_-aderenoceptor) in airways. Although activation of β_2_-aderenoceptor on airway smooth muscle induces bronchodilation, β_2_-adrenoceptor on airway epithelial cells increase mucus production, which exacerbates the symptoms of asthma and COPD [25, 37]. In contrast, it is well established that cAMP stimulates cilliary motility of airway epithelium [38–40]. These findings point out the possibility that activation of the dopamine D_1_ receptor on airway epithelium might enhance mucociliary clearance, which is beneficial for asthma and COPD patients with impaired airway mucociliary clearance. Moreover, elevation of cAMP levels and activation of PKA through G_s_-coupled receptor also contributes to bronchial epithelial wound repair and regulation of cystic fibrosis transmembrane regulator (CFTR) activity in airway epithelium [41–43]. Thus, activation of the dopamine D_1_ receptor on multiple cell types in the airway could affect multiple beneficial and potentially detrimental airway effects and it is unclear what the net effect would be in the setting of allergic lung inflammation and bronchoconstriction. A limitation of the present study is that we have used submerged NCI-H292 cells to examine dopamine D_1_ receptor-mediated MUC5AC expression. Submerged cells in culture may not recapitulate the phenotype of in vivo airway epithelium as closely as primary cultured bronchial epithelial cells grown at an air-liquid interface. Further studies are required to identify the possible diverse roles of dopamine D_1_ receptor in the normal physiology and pathophysiology of the airway.

## Conclusions

In summary, our major findings in this study are that G_s_-coupled dopamine D_1_ receptors are expressed in human airway epithelium, and stimulate cAMP production, CREB phosphorylation, and MUC5AC expression. These results combined with our previous findings [20], suggest that activation of the dopamine D_1_ receptor in airways can have complex net effects from the activation of the dopamine D_1_ receptor on multiple cell types. While activation of the dopamine D_1_ receptor on airway smooth muscle could have a bronchodilatory therapeutic benefit, the activation of the dopamine D_1_ receptor on airway epithelium could induce mucus overproduction which may be counteracted by a beneficial effect on ciliary activity.

## Abbreviations

cAMP: cyclic AMP
CFTR: Cystic fibrosis transmembrane regulator
CREB: cAMP response element binding protein
CSE: Cigarette smoke extract
HASM: Human airway smooth muscle
PBS: Phosphate-buffered saline
PKA: Protein kinase A
